# 100 years later, little has changed in Brazil: disinformation and pandemic

**DOI:** 10.4314/ahs.v21i4.52

**Published:** 2021-12

**Authors:** Silva Heslley Machado

**Affiliations:** University Center of Formiga (UNIFOR/MG) and State University of Minas Gerais (UEMG). Science and Education

## Abstract

About 100 years separate the last two pandemics that hit humanity, but scientific development does not seem to have impacted Brazilian society, including its leaders. Remedies without scientific proof, obviously without effectiveness, have been used in Brazil against the Spanish flu and nothing has changed against COVID-19. But perhaps today the process of disinformation is worse because the Internet and social networks are too efficient to spread Fake News, resulting in doctors, politicians and journalists prescribing all kinds of innocuous medicines. In this way, Brazil and its government conduct an erratic confrontation of the disease, based on scientific denialism, with tragic results.

Disinformation is widespread in Brazilian society 1, Without scientific proof, malaria drugs are widely used in Brazil against the covid global pandemic. This is driven by grassroot movements against the application of the vaccine that preach for “freedom”, the right not to vaccinate, the risks of vaccination, etc. Celebrities from politics and other circles shout conspiracy theories of all kinds that pervade the country. There is a supposed “freedom” for doctors to prescribe drugs that have no proven efficacy, but have known side effects. Public health authorities try to react, as well as academic circles, but it seems a lost battle with many victims.

Try to guess which time period I am talking about? The beginning of the 20^th^ century, or the present day? You will get it right anyway; ignorance has persisted in Brazil throughout the centuries. During the Spanish flu pandemic, the cure would be the quinine salt, a medicine used against malaria, as it happens today with hydroxichloroquine, a sad coincidence. You could also use aspirin, ammonia, salt water, smears on the skin, inhaled substances against congestion and even “Bovril” (a salty and thick meat extract) 2; as it occurs against COVID-19 in Brazil with vermifuge, antibiotics and others. In the beginning of the last century the Brazilian revolt of the vaccine occurred 3, almost ten years before the pandemic, it was necessary the force to apply them, the cost was high for the society.

It is necessary to relativize the movement named “Revolt of the Vaccine” at the beginning of the 20^th^ century by comparing it with today's anti-vaccine movements. Rio de Janeiro was going through a process of remodeling the city, with part of the population being displaced, going to live in the hills and peripheries. The city faced a smallpox epidemic in 1904 and compulsory vaccination was sought in military-style campaigns in which sanitary police with the power to enter houses to disinfect them and hunt for transmission agents such as mosquitoes and rats. In this context, the press was against the man who had assumed the equivalent of the current Ministry of Health, Oswaldo Cruz, ridiculing his actions; the scenario for the revolt was set. The result was generalized violence, on the part of the population and the authorities, with deaths, many wounded and imprisoned, and many even deported for forced labor in a distant Brazilian state. So there was a whole social web that could explain, not justify, the vaccine revolt of that time 4.

There is no social situation more prone to a social revolt than a government that is negligent with its role. If in the last century, one could consider the exaggerations of authorities, today it would be their leniency in the face of the pandemic.

However, discovery of the viruses had occurred earlier in independent studies in 1892 and 1898 by researchers studing the agent that caused a disease in plants, staining the tobacco leaves. It was named tobacco mosaic virus and become a milestone for the development of the science of virology. It was not until much later, with the invention of the electron microscope in the 1940s that viruses could be visualized and their nature better understood 5. The first reference to a disease hypothetically caused by a virus (and later confirmed) was in an article published in 1881. The Cuban Carlos Finlay realized and reported that there would be a causative agent of yellow fever, that the mosquito would only be a transmitter, and in an almost prophetic way suggested that this agent would be an “amorphous virus”^6^. Some years later, the American Walter Reed led a study that helped better understand the disease cycle^7^. However, it would take decades for the assumptions about the virus to be confirmed, and until today many people do not believe that several diseases can be caused by viruses, especially in countries that have strong dissemination of Fake News.

It is currently being deconstructed a beautiful history of science and medicine that began with the observations, experiments, and tests of the British physician, Edward Jenner^8^. When noticing that milkmaids did not become infected with human smallpox, the doctor could not imagine that, in the distant 1800s, he would be promoting a revolution in medicine and world health. Almost two centuries later the disease would be eradicated, although we still have samples in some laboratories around the world. However, in the 1970s ignorance and denialism were not in vogue and everyone in the world was vaccinated.

The result: we were free of one of the greatest historical scourges of humanity 9,10, the following generations don't even know the drama that those contaminated with smallpox suffered, with its consequences, when not mortal, very serious and deforming^11^. Without a doubt, the greatest victory of the world health organization^12^. Today, it would be difficult to eradicate AIDS, Covid-19 or Ebola, for example, because the spread of Fake News is so efficient that eradication would be almost a utopia, given the number of those who would refuse to take the vaccine.

Today, with all the knowledge acquired, the repudiation of vaccines is growing in the middle of a pandemic, while medicines without proof are prescribed by doctors^13^, journalists, and politicians^14^, especially in Brazil. The denialist doctors, who, in addition to prescribing innocuous remedies, spread their false information (deceiving the population from their social prestige), would make the pioneer British doctor feel disturbed.To make matters worse, we have the internet and social networks to spread obscurantism efficiently, which unfortunately seems to be a global problem 15.

Perhaps in Brazil this obscurantism has reached sectors that few imagined would be impacted by scientific denialism, such as health care and, notably, among doctors^16^. But it's important to observe what happens in countries like the US and France 16, for example, where governments have to nearly foce the population to get vaccinated, with awards and demands, in the face of refusal to save their lives and the lives of those around them, and we will realize the extent of the challenge posed to humanity.
